# Radiomics of the primary tumour as a tool to improve ^18^F-FDG-PET sensitivity in detecting nodal metastases in endometrial cancer

**DOI:** 10.1186/s13550-018-0441-1

**Published:** 2018-08-22

**Authors:** Elisabetta De Bernardi, Alessandro Buda, Luca Guerra, Debora Vicini, Federica Elisei, Claudio Landoni, Robert Fruscio, Cristina Messa, Cinzia Crivellaro

**Affiliations:** 1 Medicine and Surgery Department, University of Milano Bicocca, via Cadore 48, 20900 Monza, MB Italy; 2 Clinic of Obstetrics and Gynaecology, San Gerardo Hospital, via Pergolesi 33, 20900 Monza, MB Italy; 3 Nuclear Medicine Department, San Gerardo Hospital, via Pergolesi 33, 20900 Monza, MB Italy; 4 Tecnomed Foundation, University of Milano Bicocca, via Pergolesi 33, 20900 Monza, MB Italy

**Keywords:** 18F-FDG PET, Endometrial cancer, PET radiomics, Nodal stage assessment, Texture analysis

## Abstract

**Background:**

A radiomic approach was applied in 18F-FDG PET endometrial cancer, to investigate if imaging features computed on the primary tumour could improve sensitivity in nodal metastases detection. One hundred fifteen women with histologically proven endometrial cancer who underwent preoperative 18F-FDG PET/CT were retrospectively considered. SUV, MTV, TLG, geometrical shape, histograms and texture features were computed inside tumour contours. On a first group of 86 patients (DB1), univariate association with LN metastases was computed by Mann-Whitney test and a neural network multivariate model was developed. Univariate and multivariate models were assessed with leave one out on 20 training sessions and on a second group of 29 patients (DB2). A unified framework combining LN metastases visual detection results and radiomic analysis was also assessed.

**Results:**

Sensitivity and specificity of LN visual detection were 50% and 99% on DB1 and 33% and 95% on DB2, respectively. A unique heterogeneity feature computed on the primary tumour (the zone percentage of the grey level size zone matrix, GLSZM ZP) was able to predict LN metastases better than any other feature or multivariate model (sensitivity and specificity of 75% and 81% on DB1 and of 89% and 80% on DB2). Tumours with LN metastases are in fact generally characterized by a lower GLSZM ZP value, i.e. by the co-presence of high-uptake and low-uptake areas. The combination of visual detection and GLSZM ZP values in a unified framework obtained sensitivity and specificity of 94% and 67% on DB1 and of 89% and 75% on DB2, respectively.

**Conclusions:**

The computation of imaging features on the primary tumour increases nodal staging detection sensitivity in 18F-FDG PET and can be considered for a better patient stratification for treatment selection. Results need a confirmation on larger cohort studies.

## Background

Endometrial cancer is the most common gynaecological cancer in developed countries [1], with increasing incidence as the global burden of obesity worsens [2]. Prognosis of this malignancy relies upon several factors as depth of myometrium invasion, lympho-vascular space invasion and lymph node (LN) involvement, being LNs the most common site of malignancy extrauterine spread [3–5].

The surgical management for nodal stage is still controversial. Two randomized trials and meta-analysis demonstrated that pelvic lymphadenectomy had no impact on survival for patients with early-stage endometrial cancer [6–11]. To minimize treatment-related morbidity and maintain the benefit of a surgical staging, the sentinel lymph node (SLN) concept has recently received an increasing interest [12].

18F-Fluorodeoxyglucose positron emission tomography/computed tomography (18F-FDG PET/CT) has been investigated as a non-invasive staging modality. In a recent prospective multicentre study including 207 patients, it demonstrated a sensitivity of 0.65 (95% confidence interval, 0.57–0.72) [13]. Small metastatic lymph node lesions may indeed remain undetected because of limited spatial resolution and associated partial volume effects [14]. In addition, the introduction of SLN biopsy ultrastaging, able to identify micrometastatic deposits, increased false-negative PET/CT findings [15]. False positive PET/CT findings in nodal detection are instead less frequent and generally due to inflammatory states.

It has been shown in several malignancies that a proper mining of quantitative FDG uptake distribution characteristics inside tumours allows obtaining prognostic information [16–20]. The aim of this study was to apply a radiomic analysis of 18F-FDG distribution inside the primary uterine lesion to help detecting suspicious nodal metastases, for a more personalized patient care of endometrial cancer. The radiomic analysis of gynaecologic primary tumour FDG uptake to predict nodal metastases has been already proposed in a cervical cancer study by Shen et al. [21]. However, in that study, a simple correlation between radiomic-based prediction and PET visual nodal detection was made. Conversely, in our study, histological analysis was considered as gold standard.

## Methods

### Patient population and PET/CT protocol

In this monocentric retrospective study, two patient databases were considered: a first database of 86 patients (DB1) used for radiomic model definition and preliminary leave one out (LOO) testing and a second database of 29 patients (DB2) used for model testing. All DB1 and DB2 women had a histologically proven endometrial cancer and were treated at San Gerardo Hospital, Monza, between January 2009 and January 2018. All subjects underwent a 18F-FDG PET/CT scan (after signing an informed consent form), followed by surgical staging. Clinical and histological characteristics of patients are reported in Table 1. Patients were injected with 3.7 MBq/kg of 18F-FDG; PET/CT scans were performed according to the standard European Association of Nuclear Medicine (EANM) protocol [22]. DB1 patients were studied on two PET/CT scanners (33 on Discovery ST and 43 on Discovery 600, GE Healthcare Milwaukee, WI, USA), using the same acquisition/reconstruction protocol: 3 min acquisition; reconstruction with ordered subset expectation maximization (OSEM), 2 iterations, 16 subsets on a voxel grid on 2.73 × 2.73 × 3.27 mm^3^; and post-filtering with a 5-mm filter in the transaxial plane and with weights 1, 4 and 1 along the axial direction. Eight DB2 patients were studied on Discovery 600, with the same acquisition/reconstruction protocol used in DB1. Twenty-one DB2 patients were instead studied on a third scanner (Discovery IQ, GE Healthcare Milwaukee, WI, USA) with different parameters: 1.5 min acquisition; reconstruction with OSEM, 6 iterations, 12 subsets with Point Spread Function modelling, on the same voxel grid of 2.73 × 2.73 × 3.27 mm^3^; and post-filtering with a-6.4 mm filter in the transaxial plane and with weights 1, 4 and 1 along the axial direction. All patients underwent surgical treatment including peritoneal cytology, total hysterectomy, bilateral salpingo-oophorectomy and surgical nodal status assessment (lymphadenectomy ± sentinel node biopsy). Nodal status at histology was considered as the standard reference for nodal involvement. The standard PET diagnosis of lymph node involvement was based on visual evidence of pathological tracer uptake at lymph node sites identified on CT images [23].

**Table 1:** Characteristics of DB1 and DB2 patient population

	DB1 (*n* = 86)	DB2 (*n* = 29)
Age (mean, range)	(66, 27–86)	(63, 30–80)
Grade		
G1	12	4
G2	38	11
G3	36	14
Histology		
Endometrioid	69	23
Clear cell/serous/mixed	12	5
Malignant mixed mesodermal tumour	5	1
Myometrial invasion		
< 50%	49	10
> 50%	37	19
LN metastases (histology)		
Yes	16	9
No	70	20
Staging FIGO		
I	55	19
II	7	1
III	23	9
IV	7	0
Adjuvant treatment		
Chemotherapy/RT	38	17
No	48	12
PET LN detection rate		
TP	8	3
TN	69	19
FP	1	1
FN	8	6

### Radiomic analysis of primary uterine lesion 18F-FDG uptake

**Fig. 1:**
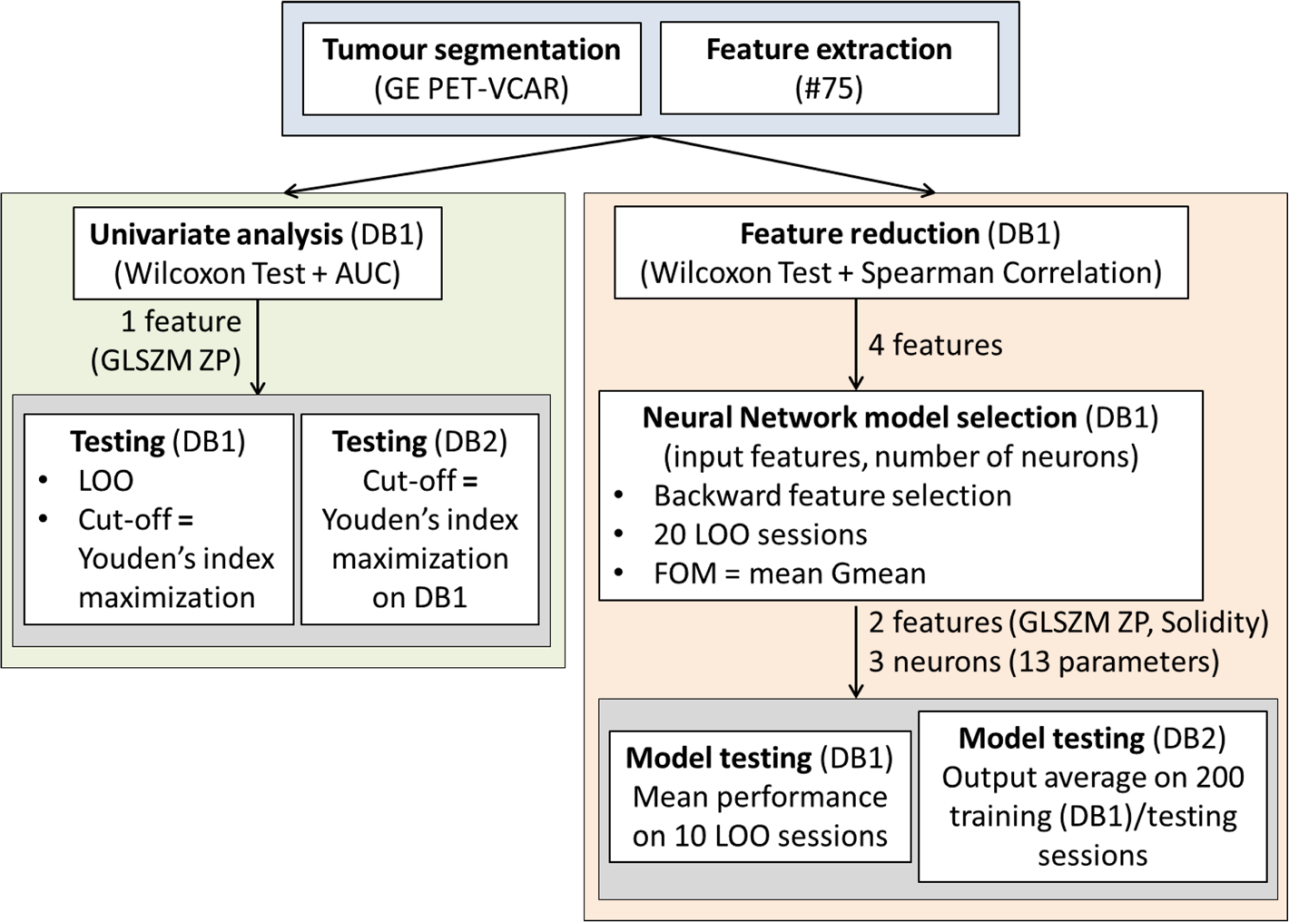
Complete pipeline of the performed radiomic analysis

A radiomic model for the prediction of lymph node involvement based on a quantitative analysis of the FDG uptake inside the primary lesion was developed and assessed. A scheme of the followed pipeline is shown in Fig. 1.

#### Tumour segmentation and feature extraction

Endometrial primary tumours were contoured with the iterative thresholding algorithm implemented in PET-VCAR software (GE Healthcare Milwaukee, WI, USA). On each volume of interest (VOI), 75 features were computed: SUVmax, SUVmean, metabolic tumour volume (MTV) and total lesion glycolysis (TLG); 6 geometrical shape features; 7 first-order features based on the grey level histogram; and 58 texture features. As to texture features, the freely available CGITA software [24] was used. Grey levels inside each VOI were resampled in *N* = 64 quantization levels [25] and nine texture matrices were computed in 3D with a 26-voxel connectivity: grey level co-occurrence matrix (GLCM) and normalized grey level co-occurrence matrix (NGLCM) [26–28]; voxel alignment matrix (VAM) [29]; grey level size zone matrix (GLSZM) [29, 30]; neighbourhood grey tone difference matrix (NGTDM) [31]; texture spectrum (TS) [32]; texture feature coding matrix (TFCM) and texture feature coding co-occurrence matrix (TFCCM) [33]; and neighbourhood grey level dependence matrix (NGLDM) [34]. Texture features were computed on these texture matrices.

#### Univariate analysis

Univariate analysis for association between features and LN metastases was performed by means of Wilcoxon rank sum test and area under the ROC curve (AUC) computation. For the feature most related with nodal involvement (i.e. the one with the smallest *P* value), an optimal cut-off was obtained by means of ROC analysis and Youden’s index maximization. The feature stratification ability was assessed on DB1 with LOO (i.e. for each excluded patient, the cut-off was defined on the remaining 85 patients) and on DB2 with the optimal cut-off defined on whole DB1.

#### Multivariate analysis: feature reduction and model selection

Correlation between features was investigated using Spearman rank correlation since relationships are non-linear and variables not normally distributed. The pool of image features for multivariate analysis (i.e. feature reduction) was obtained by first selecting the feature with the smallest univariate *P* value and the largest AUC and by subsequently adding features with increasing *P* values (if ≤ 0.01) only if characterized by an absolute value of the Spearman rank correlation < 0.85 vs already selected features.

A neural network classifier with one hidden layer of neurons was used. Model selection implies the choice of the number of inputs (among the pool of features previously selected) and the choice of the hidden layer neuron number. A stepwise backward feature selection scheme was adopted, and for each group of input features, neural networks with 2, 3, 4 and 5 hidden layer neurons were assessed. Each model was evaluated by means of the mean gmean index (square root of product between sensitivity and specificity) on 20 LOO sessions on DB1. In each session, the 85 patients were randomly divided in training set (72 patients) and validation set (13 patients), paying attention to maintain the positive lymph node percentage found in DB1 (23%) in both sets. The positive case weight during training was quadruplicated in order to compensate for sample imbalance. Features given in input to neural networks were all normalized in the range [− 1 1]; neural network weights and biases were randomly initialized.

#### Multivariate analysis: model testing

The selected multivariate model (i.e. neural network with the selected input features and the selected hidden layer neuron number) was firstly assessed on DB1 by means of 20 LOO sessions. Mean and standard deviation of sensitivity and specificity were computed. The neural network was then trained 200 times on whole DB1 (following the rules for subdivision in training and validation sets described in the ‘Multivariate analysis: feature reduction and model selection’ section) and then tested on DB2. The average of the 200 session outputs was taken as model output.

#### Construction of an unified prognostic framework

**Fig. 2:**
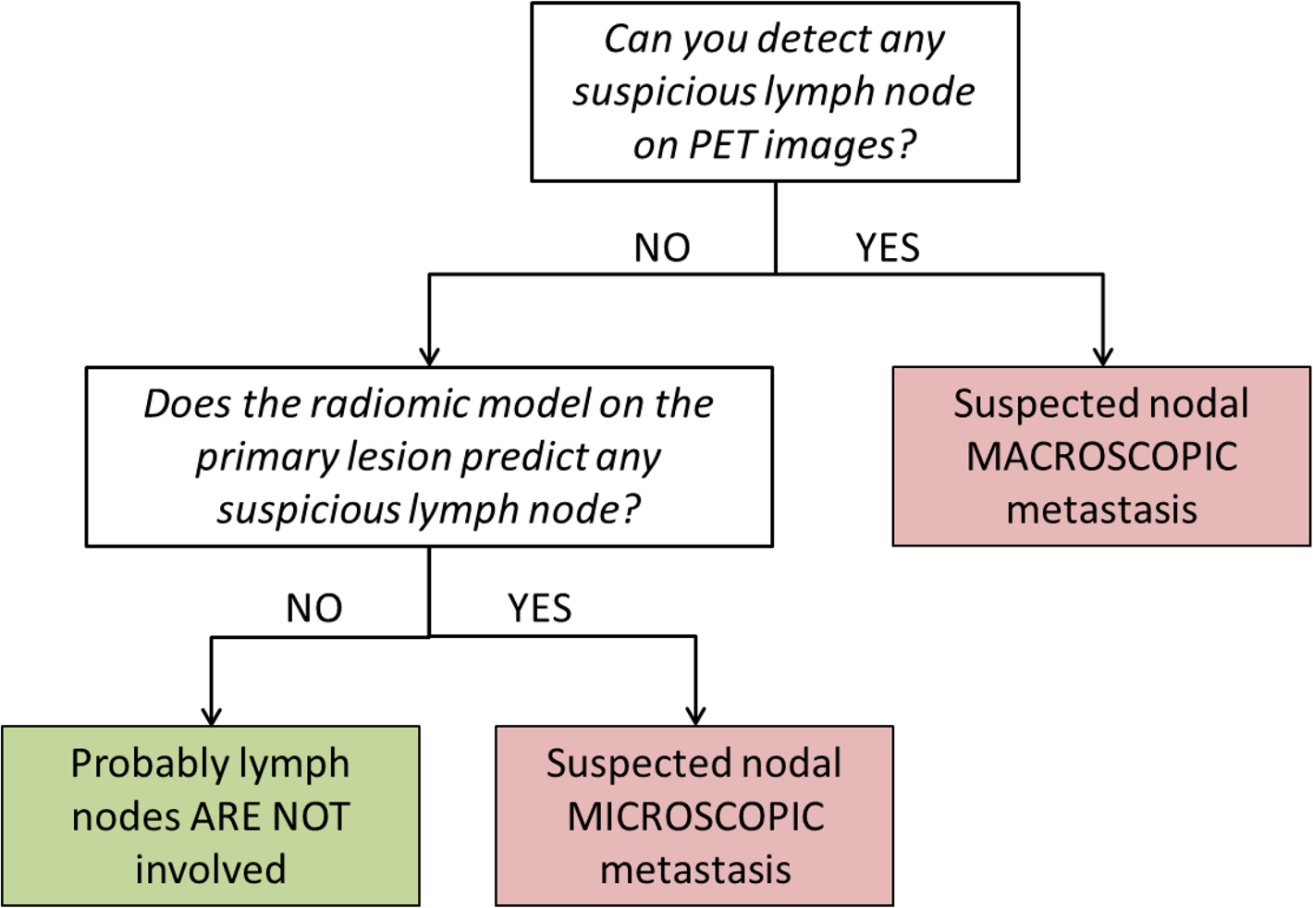
Unified prognostic framework combining lymph node visual assessment and radiomic primary lesion analysis

The results of lymph node visual assessment and radiomic analysis were combined together into a unified prognostic framework for nodal involvement detection, represented in Fig. 2. The unified framework was assessed following the same scheme used for radiomic model testing.

## Results

On the 86 DB1 patients, the prevalence of nodal metastases was 23%; FDG PET/CT sensitivity and specificity in detecting nodal metastases were respectively 50% and 99%. On the 29 DB2 patients, the prevalence of nodal metastases was 45%; FDG PET nodal metastases detection sensitivity and specificity were respectively 33% and 95%. Results are reported in Table 1.

### Radiomic analysis of primary uterine lesion 18F-FDG uptake

#### Univariate analysis

**Fig. 3:**
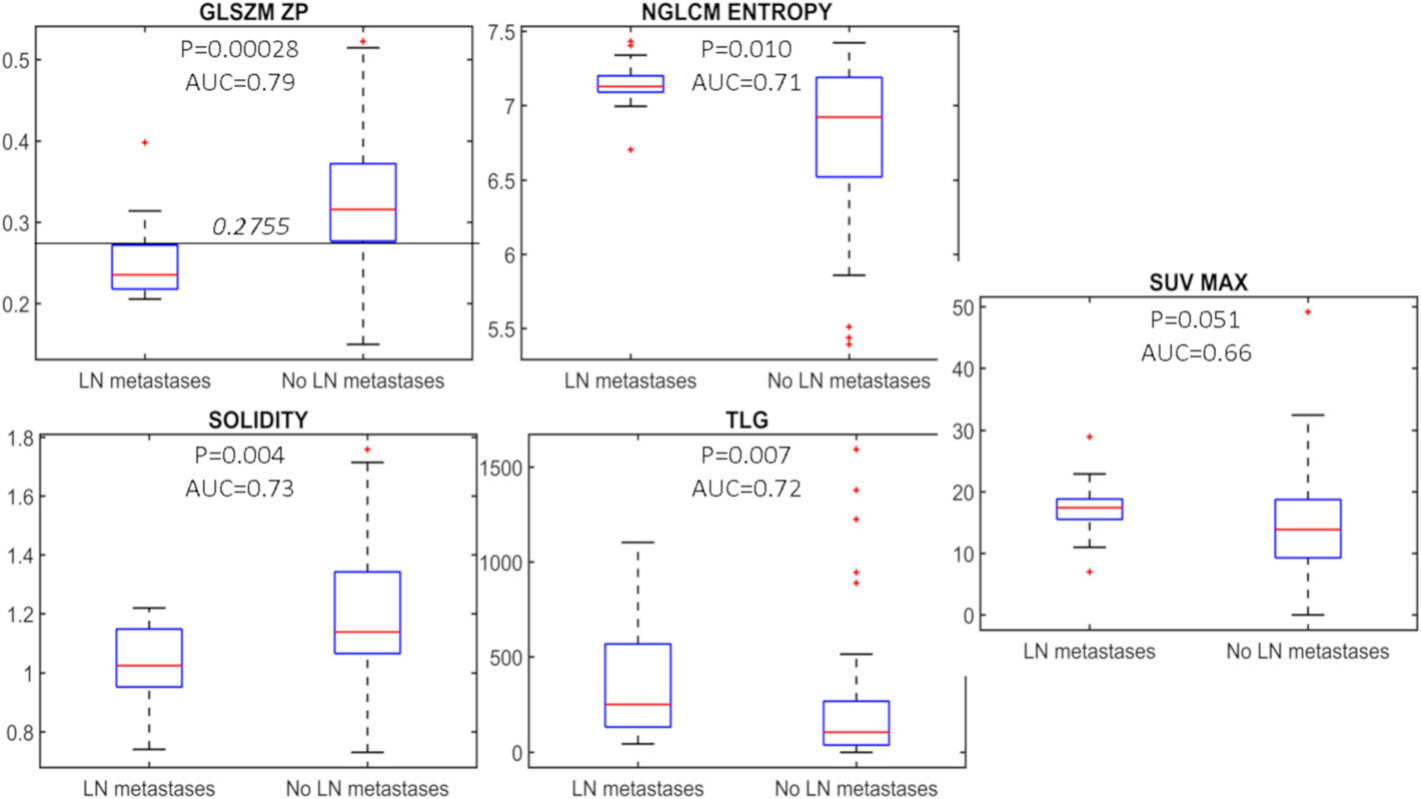
Boxplot representation of the four features selected after feature reduction to classify patients with and without LN metastases. *P* values of univariate test and AUC values are reported. SUVmax distribution is added for completeness

In the univariate analysis, the widely used SUVmax was not significant (Fig. 3). Twelve features (TLG, two geometrical features, two GLCM features, two NGTDM features, two GLSZM features, one NGLCM feature, one NGLDM feature) were instead able to differentiate patients with LN metastases with a *P* value ≤ 0.01. The lowest *P* value and the highest AUC were obtained by GLSZM ZP (zone percentage of GLSZM). GLSZM in each sample (*n*, *s*) contains the number of 3D zones of size *s* and grey level *n* in VOI. Features computed on GLSZM provide a characterization of regional tumour heterogeneity, i.e. describe variations of intensity between regions and variations of homogeneous area size [35]. Zone percentage of GLSZM is the ratio between the total number of zones and the number of voxels in VOI. Tumours with nodal metastases show a reduced GLSZM ZP if compared with tumours without nodal metastases, with a *P* value of 2.8 × 10^−4^ and an AUC of 0.79.

#### Multivariate analysis: feature reduction and model selection

Feature reduction for multivariate analysis was applied to the 12 features with *P* ≤ 0.01, and 3 further features were selected: TLG, solidity (ratio between VOI volume and VOI convex hull volume, smaller for VOIs presenting concavities or surface irregularities) and NGLCM ENTROPY (Entropy of NGLCM). NGLCM is a *N* × *N* matrix, where *N* is the number of grey levels in VOI after quantization (*N* = 64 in our implementation). NGLCM (*i*, *j*) counts the relative frequency of grey levels *i* and *j* at a one voxel distance. NGLCM features therefore characterize tumour local heterogeneity on the basis of grey levels variations between neighbouring voxels. Entropy in particular measures the randomness of grey level distributions [28]. Spearman rank correlation vs GLSZM ZP of TLG, solidity and NGLCM ENTROPY is − 0.65, 0.83 and − 0.49, respectively. In Fig. 3, the distribution of the four selected features in DB1 patients with and without LN metastases is displayed, together with corresponding univariate analysis *P* values and AUC values. For GLSZM ZP, the optimal cut-off defined on DB1 (0.2755) is also represented. As to model selection, the best results were obtained with a 13 parameter neural network with three hidden layer neurons and 2 inputs (GLSZM ZP and SOLIDITY).

**Fig. 4:**
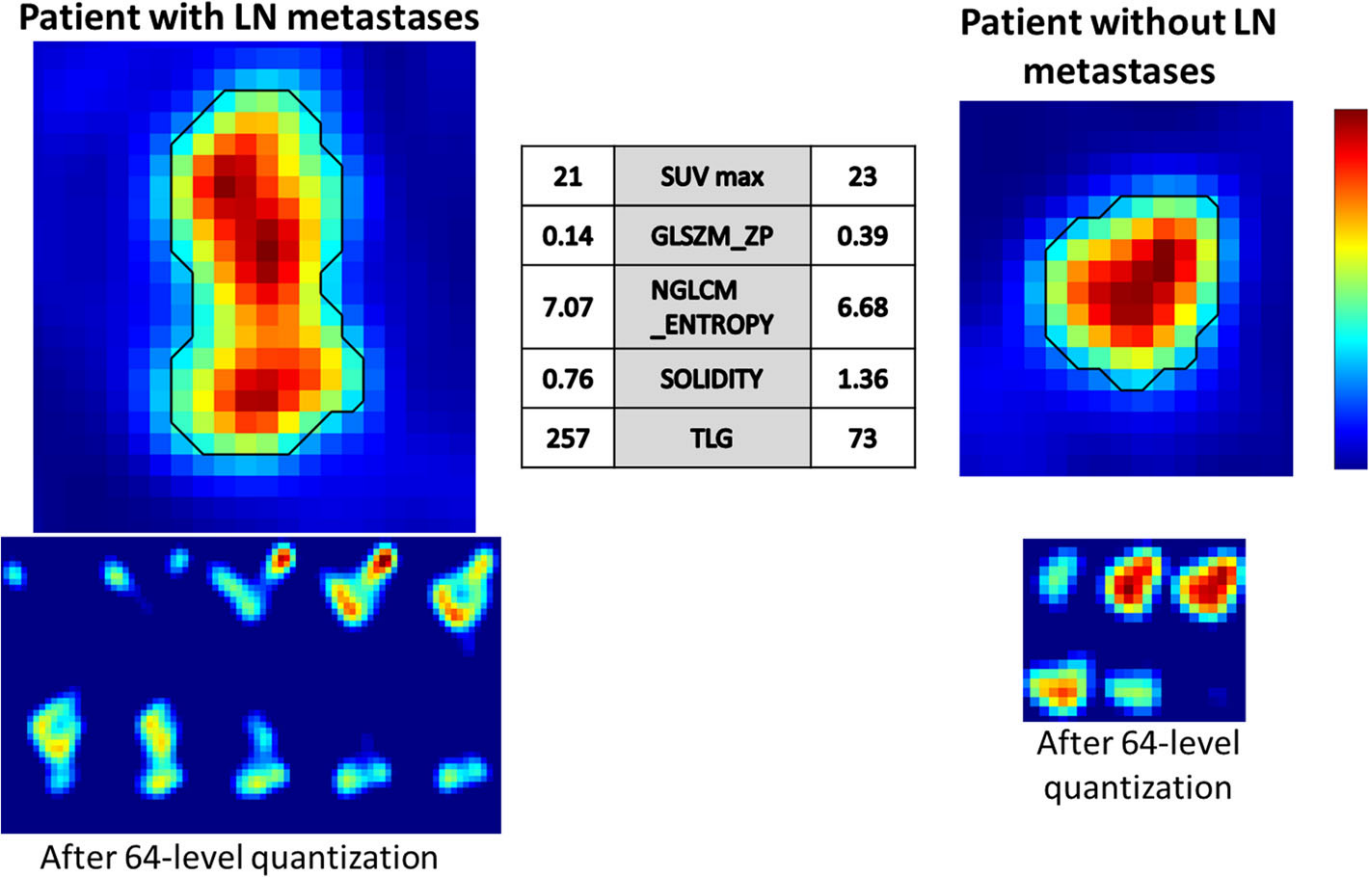
Example of endometrial tumour with LN metastases (left panel) and endometrial tumour without LN metastases (right panel). Feature values computed on the tumours are displayed in the central table

In Fig. 4, two examples of endometrium tumours (the left one with LN metastases, the right one without LN metastases) are represented, together with the relevant feature values and SUV max values. GLSZM ZP practically quantifies the number of uniform uptake regions inside the lesion after 64 level quantization, i.e. after the subdivision of the whole range of lesion uptake values into 64 equally spaced intervals. Lesions with lower GLSZM ZP, like the left one in Fig. 4, are the ones characterized by a wider range of uptake values before quantization (e.g. by the co-presence of very high-uptake voxels and very low-uptake regions, like necrotic areas) so that, after quantization, the number of uniform areas inside the lesion results small. Conversely, lesions with higher GLSZM ZP, like the right one in Fig. 4, are those characterized by a more uniform uptake and so by a narrower uptake value range, resulting in a higher number of uniform areas after quantization.

#### Radiomic model testing

Results of univariate and multivariate model testing on DB1 (LOO) and DB2 are shown in Table 2, together with the results of the nodal status visual assessment and the results of the unified prognostic framework combining nodal status visual assessments and primary tumour radiomic analysis (described in Fig. 2).

**Table 2:** Results of univariate and multivariate model testing on DB1 (LOO) and DB2, together with results of nodal status visual assessment and unified prognostic framework

DB1-LOO (#86)		LN visual detection	Univariate model (GLSZM ZP)	LN visual detection + univariate model	Multivariate model (GLSZM ZP + Solidity)	LN visual detection + multivariate model
	Sensitivity	50%	75%	94%	67% ± 8%	86% ± 6%
	Specificity	99%	81%	67%	68% ± 3%	66% ± 3%
DB2 (#29)		LN visual detection	Univariate model (GLSZM ZP)	LN visual detection + univariate model	Multivariate model (GLSZM ZP + Solidity)	LN visual detection + multivariate model
	Sensitivity	33%	89%	89%	89%	89%
	Specificity	95%	80%	75%	80%	75%

**Fig. 5:**
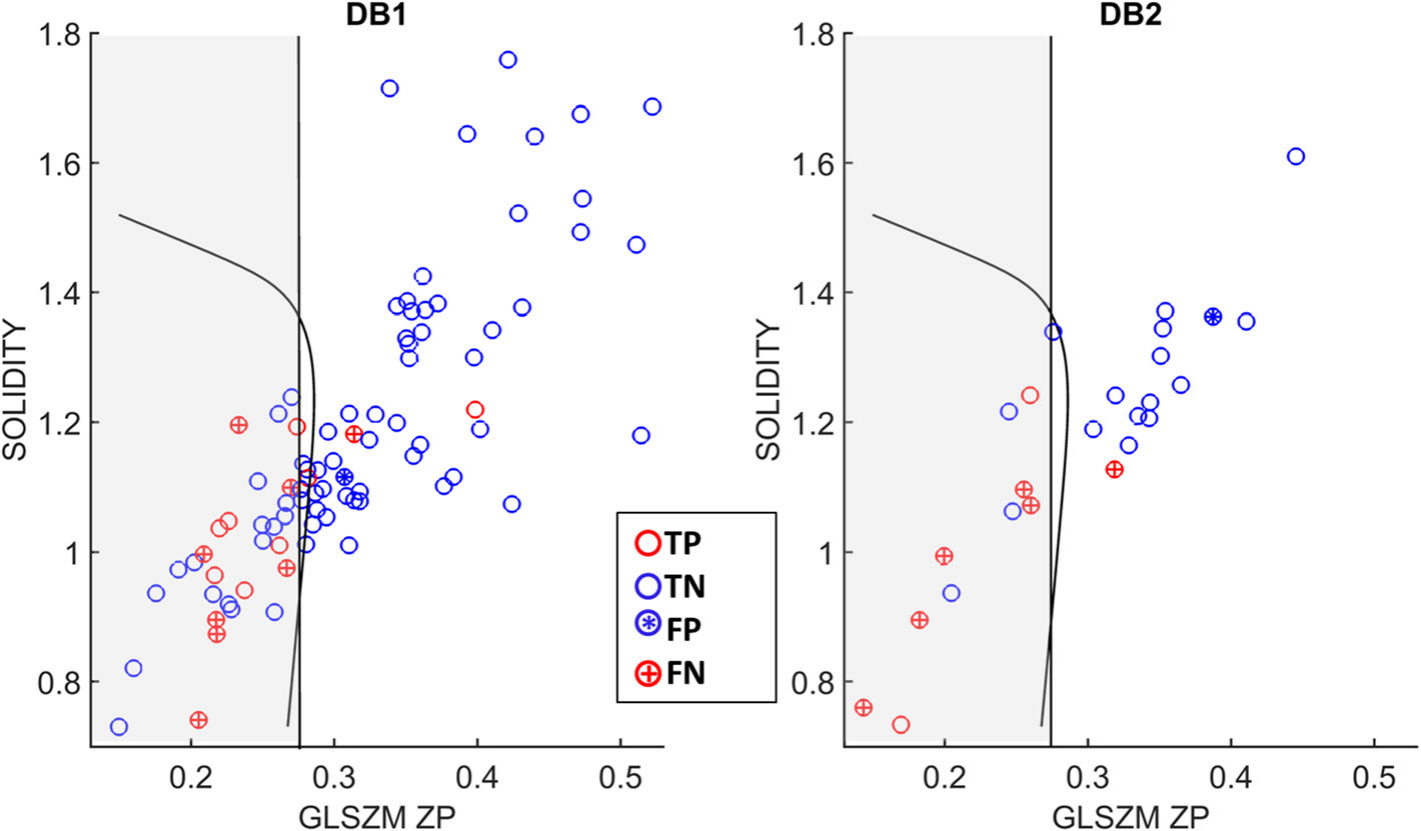
Results of LN visual detection and radiomic analysis are represented together on DB1 (left panel) and DB2 (right panel). For each patient, GLSZM ZP and SOLIDITY values are shown. Blue circles correspond to patients with histologically negative lymph nodes, while red circles to patients with histologically positive lymph nodes. Patients with a ‘+’ in the red circle and with a ‘*’ in the blue circle are those which are misclassified by the LN visual detection: in particular, blue circles containing a ‘*’ are false positives at LN visual detection, while red circles containing a ‘+’ are false negatives. The grey area contains patients classified as positive by the univariate radiomic analysis (GLSZM ZP < 0.2755); therefore false positives are blue circles in the grey area, while false negatives are red circles in the white area. The curve line is an example of neural network trained on the 86 DB1 patients

In Fig. 5, results obtained on all DB1 (left panel) and DB2 patients (right panel) are shown.

The analysis of Fig. 5 and Table 2 shows that LN visual detection and primary tumour radiomic analysis are complementary. In particular, there are patients with negative LN at visual detection which have very low GLSZM ZP values and so are classified as positive by the radiomic analysis. The performance of visual analysis is worse on DB2 than on DB1, while radiomic analysis (trained on DB1) performs better on DB2. This could be explained by the increased incidence of micrometastases in DB2 (24% vs 13% in DB1). The combination of LN visual detection and radiomic analysis increases sensitivity compared to LN visual detection alone; however, since patients classified as LN positives by one of the two approaches are globally classified as positive, false positive findings augment, thus reducing specificity. The neural network multivariate model performs equally (on DB2) or even worse (on DB1) than the univariate model relying on GLSZM ZP only.

## Discussion

American College of Radiology Appropriateness Criteria suggest 18F-FDG PET/CT as the best technique for endometrial cancer nodal staging (score = 9), in particular for high-risk histologies. However, PET spatial resolution limits make visually detectable only LN metastatic deposits larger than 5 mm, resulting in FN findings. The PET/CT FN rate has recently further increased, due to sentinel node biopsy and ultrastaging improvement, able to identify micrometastases (< 2 mm) and isolated tumour cells, not detectable at PET/CT scans [15]. The aim of this study was therefore to assess if a radiomic approach on the primary uterine lesion could improve 18F-FDG PET sensitivity for nodal metastases. Standard imaging features like SUV, MTV and TLG were taken into account, together with histogram-based features, texture features and geometrical shape features. Data acquired and reconstructed on three different scanners were considered, with the specific aim of finding a nodal involvement predicting model with a certain robustness degree. It is worth noticing that the reconstruction voxel size was maintained identical in the three scanners, since it is known to be the factor most influencing texture feature absolute and prognostic values.

SUVmax, the most widely used PET feature, considered as an important indicator reflecting tumour aggressiveness, such as myometrial invasion or tumour grade, was not significantly correlated to lymph node status according to previous studies [36, 37]. Tumours with LN metastases were conversely generally characterized by higher MTV and higher TLG, as already observed in our previous report [23], and even more by higher heterogeneity and irregular borders, thus confirming the poorer prognosis of tumours presenting these characteristics, as already observed in PET radiomic literature.

An univariate prediction model relying on a unique heterogeneity feature (GLSZM ZP) and a neural network multivariate model considering GLSZM ZP and a geometrical shape feature (SOLIDITY) were defined on a database of 86 patients (DB1) and successively assessed on DB1 with a LOO approach and on a second independent database of 29 patients (DB2). On DB1, where sensitivity and specificity of LN visual detection were 50% and 99%, the univariate model obtained a sensitivity of 75% and a specificity of 81%, while the multivariate model a sensitivity of 67% ± 8% and a specificity of 68% ± 3% on 20 LOO sessions. On DB2, where sensitivity and specificity of LN visual detection were instead 33% and 95%, univariate and multivariate models performed identically, achieving a sensitivity of 89% and a specificity of 80%.

Our results show that, for nodal staging, GLSZM ZP alone performs better than any other feature or multivariate model. GLSZM ZP is a regional texture feature whose ability in differentiating patients with different prognosis has been already observed in various tumours [38]. Tumours characterized by the co-presence of high-uptake and low-uptake areas (and so by heterogeneous content) have a lower GLSZM ZP value and a poorer prognosis. In DB1, GLSZM ZP correlates with LN metastases presence with *P* = 2.8 × 10^−4^. GLSZM ZP has already been shown to be reproducible in test-retest studies [35], robust vs. segmentation algorithms, reconstruction parameters and algorithms [17, 19, 39]. The robustness of GLSZM ZP is here confirmed. Models were indeed defined on DB1 patients, which were studied on Discovery 600 and Discovery STE GE scanners, and validated on DB2 patients, which were instead for the most part (21/29) studied on a Discovery IQ GE scanner with a different acquisition/reconstruction protocol. In particular, Discovery 600 and Discovery ST patients were reconstructed without PSF modelling, while Discovery IQ patients with PSF modelling, which is known to influence image texture appearance. GLSZM ZP appears therefore particularly robust vs this aspect. The independency of GLSZM ZP from the scanner (together with that of Solidity, MTV, TLG, SUVmax and SUVmean) was also a-posteriori verified by means of a Kruskal-Wallis test.

In this study, we propose to combine a radiomic prediction model and the results of LN metastases visual detection into a unified framework to improve PET technique sensitivity. On DB1, the unified framework obtained a sensitivity of 94% and a specificity of 67%, while on DB2 a sensitivity of 89% and a specificity of 75%. A joint model of this kind maximizes the exploitation of the PET technique information for a more personalized and effective surgical treatment selection. The combination of PET/CT and SLN mapping has been proposed to minimize complications and to maximize LN status and cure rate definition [15]. The high PPV of FDG PET allows to direct patients positive at LN visual detection to lymphadenectomy, with debulking aim. However, in case of negative PET, microdisease cannot be excluded. Radiomics can help in further stratifying the risk of nodal metastases, to better select women who can benefit from SLN procedure and ultrastaging, for resource optimization. Negative PET patients with low GLSZM ZP seem in fact more likely to have micrometastases and should therefore be referred to SLN biopsy and ultrastaging in third level specialized hospitals.

Our preliminary results are promising in order to gather as much information as possible from an examination that is necessary in clinical setting of endometrial cancer patients. GLSZM ZP can be easily extracted from PET images by means of many freely available radiomic tools. Future perspectives will include the further assessment of the predictive values of CT/MRI features and eventually the construction and validation of multimodal models to further exploit PET/CT technique potentialities.

The main limitations of the present study are the small number of patients, in particular in DB2. In addition, DB2 is more recent and all patients underwent to SLN biopsy and ultrastaging, able to identify micrometastatic deposits not detectable by older histological techniques and largely under the PET spatial resolution limit [40]. This may justify the higher rate of micrometastases (24% vs 13%), the lower sensitivity of LN visual detection (33% vs 50%) and the better performance of the radiomic analysis observed in DB2 with respect to DB1. We plan to collect new data for training and new independent data for testing to confirm trends observed on DB2. Furthermore, we hope that these preliminary data could encourage cooperative efforts to confirm or to reject results on a wider and therefore more significant patient cohort of endometrial cancer patients.

The objective of this work was to try to improve PET sensitivity for nodal staging. A further step for treatment definition improvement may concern the insertion of PET features and laboratory/histological parameters into a global multivariate staging model. On the 115 patients we have, we have verified that, in accordance with literature, myometrium invasion and lympho-vascular space invasion are significantly correlated with LN metastases presence. These two variables may therefore be significant covariates in a global staging model. Grade and histology were instead not correlated. It is worth noticing however that most of patients in our database have endometrioid histology; thus, the correlation with histology may be assessed on a larger and more heterogeneous sample.

## Conclusions

Endometrial cancers with LN metastases were generally characterized by higher heterogeneity at PET scan, and imaging features computed on the primary tumour were able to improve PET sensitivity in LN metastases detection.

## Abbreviations

18F-FDG: 18F-Fluorodeoxyglucose
AUC: Area under the ROC curve
CT: Computed tomography
DB1: First database of 86 patients
DB2: Second database of 29 patients
EANM: European Association of Nuclear Medicine
FN: False negative
FP: False positive
GLCM: Grey level co-occurrence matrix
GLSZM ZP: Zone percentage of GLSZM
GLSZM: Grey level size zone matrix
LN: Lymph node
LOO: Leave one out
MRI: Magnetic resonance imaging
MTV: Metabolic tumour volume
NGLCM ENTROPY: Entropy of NGLCM
NGLCM: Normalized grey level co-occurrence matrix
NGLDM: Neighbourhood grey level dependence matrix
NGTDM: Neighbourhood grey tone difference matrix
OSEM: Ordered subset expectation maximization
PET: Positron emission tomography
SLN: Sentinel lymph-node
SUV: Standardized uptake value
SUVmax: Standardized uptake value maximum
SUVmean: Standardized uptake value mean
TFCCM: Texture feature coding co-occurrence matrix
TFCM: Texture feature coding matrix
TLG: Total lesion glycolysis
TN: True negative
TP: True positive
TS: Texture spectrum
VAM: Voxel alignment matrix
VOI: Volume of interest
